# Cribellate thread production as model for spider’s spinneret kinematics

**DOI:** 10.1007/s00359-020-01460-4

**Published:** 2021-01-23

**Authors:** Margret Weissbach, Marius Neugebauer, Anna-Christin Joel

**Affiliations:** 1 Institute for Biology II, RWTH Aachen University, Worringerweg 3, 52074 Aachen, Germany; 2 Department of Biological Sciences, Macquarie University, Sydney, Australia

**Keywords:** Cribellate capture thread, Functional morphology, Biomimetic, Fibre processing, Spigot

## Abstract

Spider silk attracts researchers from the most diverse fields, such as material science or medicine. However, still little is known about silk aside from its molecular structure and material strength. Spiders produce many different silks and even join several silk types to one functional unit. In cribellate spiders, a complex multi-fibre system with up to six different silks affects the adherence to the prey. The assembly of these cribellate capture threads influences the mechanical properties as each fibre type absorbs forces specifically. For the interplay of fibres, spinnerets have to move spatially and come into contact with each other at specific points in time. However, spinneret kinematics are not well described though highly sophisticated movements are performed which are in no way inferior to the movements of other flexible appendages. We describe here the kinematics for the spinnerets involved in the cribellate spinning process of the grey house spider, *Badumna longinqua*, as an example of spinneret kinematics in general. With this information, we set a basis for understanding spinneret kinematics in other spinning processes of spiders and additionally provide inspiration for biomimetic multiple fibre spinning.

**Supplementary Informtion:**

The online version of this article (10.1007/s00359-020-01460-4) contains supplementary material, which is available to authorized users.

## Introduction

Spider silk captivates researchers for many decades now, not least because of its extraordinary material properties (Vollrath 2000; Eisoldt et al. 2011). One of the most studied characteristics to uncover the principle behind the high toughness of spider silk is its molecular structure, which enhances toughness by a combination of elastic and crystalline domains in the protein (Termonia 1994; Hayashi et al. 1999; Becker et al. 2003; Mortimer and Holland 2015). However, there is not a single type of spider silk: Spiders possess up to nine different silk glands, each of which produces a specific type of silk with equally distinct properties (Kovoor and Peters 1988; Coddington 1989; Vollrath 1992). These different types of silk are used for various purposes and can even be combined, e.g., to a spider’s web or attachment discs (Foelix 2011). To anchor their threads to a substrate, e.g., spiders specifically combine several fibre types, influencing the mechanical properties of the final attachment disc by this interweaving (Wolff et al. 2015). However, the most complex combination of different silk types to a single thread can be observed in the assembly of capture threads of cribellate spiders. With these threads, cribellate spiders catch their prey without the use of viscous glue. By involving nanofibres, adhesion is achieved by a combination of van der Waals and capillary forces, through which the cuticular waxes of the prey are absorbed (Hawthorn and Opell 2002; Bott et al. 2017). Up to six different types of silk, including thousands of nanofibres (Ø 10–30 nm) and several larger fibres, are connected to complex three-dimensional structures (Peters 1992; Eberhard and Pereira 1993; Opell 2002; Joel et al. 2015; Grannemann et al. 2019). The use of different fibres influences the mechanical properties of the capture threads beyond the molecular properties of their individual silks, comparable to a fibre-composite (Michalik et al. 2019; Piorkowski et al. 2020).

The silk fibres are extruded from spigots on the spinnerets of a spider. Spinnerets are segmented appendages on the opisthosoma of a spider (Mariano-Martins et al. 2020). They are articulated to the body, and their segments are connected to each other, via movable joints. Most of the spiders possess three pairs of spinnerets: anterior lateral (ALS), posterior median (PMS), and posterior lateral (PLS) spinnerets (Montgomery 1909; Glatz 1972, 1973; Platnick et al. 1991). In addition, cribellate spiders possess a further spinning organ, the cribellum (Cr). It can be described as a transverse and moveable structural unit covered with spigots which is situated anteriorly of the other spinnerets (Blackwall 1839; Bertkau 1882; Montgomery 1909). In contrast, some ancient spiders, such as the Mesothelae, possess a further pair of spinnerets, the anterior median spinnerets, at the corresponding position of the cribellum in cribellate spiders. This fourth pair of spinnerets as well as the cribellum embryologically develop from the same primordia, which implies a homology of both structures, thus, possibly featuring similar manoeuvrability (Pechmann et al. 2010; Foelix 2011). Previous studies of the cribellate capture thread production already proved elaborate manoeuvrability of the spinnerets coupled with a highly coordinated sequence of movements in three-dimensional space to create such complex structures (Joel et al. 2015; Grannemann et al. 2019). The mobility of the spinnerets is achieved by muscles located at different attachment sites (Glatz 1972, 1973; Magalhães et al. 2017). By an increase of haemolymph pressure, some movements can be controlled by a hydraulic mechanism, as described for the movement of legs (Kropf 2013; Labarque et al. 2017). Interestingly, apart from some general descriptions of movements and spinneret morphologies, hardly anything is known about the kinematics of spinnerets. However, the morphology of the spinnerets coupled with their spatial movements is crucial for the accessibility of different spigots extruding the specific fibres and thus the interweaving of fibres (Eberhard 2010).

Though the evolutional origin of the spinnerets is still open to discussion, several facts hint to originally locomotor appendages which lost their primary function (Shultz 1987; Hilbrant 2008; Pechmann and Prpic 2009). It is conspicuous that spinnerets have a segmentation and complex musculature and thus achieve a flexibility that is in no way inferior to that of the legs (Blackledge et al. 2011). There are a few works on the kinematics of spider legs describing the locomotor system via movement radii, length ratios, and co-ordinates (Ehlers 1939; Weihmann et al., 2010; Foelix 2011). But in contrast to the legs, the spinnerets interact with each other and can create three-dimensional structures. Hence, we aim to analyse the spinneret kinematics using the cribellate spinning process, in which almost all spinnerets are involved in the process and a highly repetitive three-dimensional structure is created as a thread. We investigated the spinneret kinematics of the synanthropic cribellate grey house spider *Badumna longinqua* (Desidae). By solving the complex choreography and uncovering conserved conditions of the process, we hope not only to unravel the general spinneret kinematics, but also to inspire future biomimetic application for complex small-scale fibre spinning techniques.

## Material and methods

### Study animals

Specimens of *Badumna longinqua* (L. Koch, 1867) were collected in public parks in Eastern Australia (Sydney (NSW), Brisbane (QLD)) and kept separately in plastic boxes with a roughened surface at Macquarie University, time-shifted by 12 h. Once a week spiders were fed with *Drosophila* *melanogaster* and water was provided once to twice per month by sprinkling the enclosure. Additionally, spiders were exported (permission no. PWS2019-AU-000248) to Aachen (Germany) and kept at elevated room temperature (~ 26 °C), room humidity (30%), and northern European diurnal rhythm. They were fed with juvenile *Acheta domestica* or *Callosobruchus maculatus*. Water was provided as described above.

### Video recordings

The spinning apparatus was studied during cribellate thread production using a video camera (Sony Alpha 6300), equipped with a 4 × magnification lens of a microscope and stabilized with a magic arm (Manfrotto) at Macquarie University. Videos were recorded with 120 fps under either white light or red light (less disturbance of night-active spiders) with a 12 h shifted day–night cycle. Recordings were performed from different perspectives, if possible from a ventral and lateral view. Video sequences always covered several spinning cycles. Additionally, the general mobility of the spinnerets was recorded using a high-speed microscope (Keyence VW-600C) with the corresponding Software (Keyence VW-9000 Motion Analyzer, Version 1.4.0.0) at a frame rate of 60–250 fps. For this purpose, the spiders were first anaesthetised with diethyl ether and then trapped using minutiae on a preparation dish to immobilise the body and legs.

### Scanning electron microscopy

Of the recorded individuals, either the exuvia were collected or living specimens were placed in the freezer (-20 °C) until they were frozen. Frozen specimens were then immediately transferred to ethanol (70%) and subsequently dehydrated over a series of increasing concentrations of ethanol (80, 90, 95, and 100%). Following, the samples were dried by hexamethyldisilazane (HMDS, ≥ 98%) in increasing concentrations (ethanol: HMDS; 3:1, 1:1, 1:3, 0:1). The concentration step 0:1 was repeated two times. The liquid was then removed, and the samples were air-dried in a fume cupboard. Before examination using a scanning electron microscope (SEM 525 M; Philips AG), samples were sputter-coated with a 10 nm layer of Au (Hummer Technics Inc.).

### Data analysis

Abstracted models of the spinnerets were created using SEM images (Online Resource 4), video stills, and the software Inkscape (Version 1.1, Inkscape Community). Measurements of the length of spinnerets and legs were performed on SEM images using a combination of the software Shotcut (Version 20.02.17), GIMP (GNU Image Manipulation Program, Version 2.10.18), and Snipping Tool (Version 10.0.18363, Microsoft Corporation). The resulting data were used as a reference for further distance measurements within the videos. Movements of structures involved in the spinning process could thus be converted into velocities over distances covered within a certain period of time. Velocities were determined on three combing cycles (N = 3) of one individual (n = 1) and listed as means. Data acquisition and statistical analysis were performed using Microsoft® Office Excel® 2020 (Version 16.0.12527.20612, Microsoft Corporation).

### Characterization of the capture thread

For SEM examinations, capture threads of *B. longinqua* were transferred to sample holders with cut-to-size conductive foil, enabling the thread to hang free between the foil strips. Care was taken to ensure an undamaged and non-stretched transfer of the threads in order to obtain a sample as representative as possible. The samples were examined without sputtering. Cribellate threads without coating charge up quickly in the SEM and can therefore only be recorded with low contrast, but unlike sputtered samples, they retain their original shape and do not collapse (Joel and Baumgartner 2017). Thus, internal fibre types can be identified in the loose mass of nanofibers.

### Preface on kinematics

**Fig. 1:**
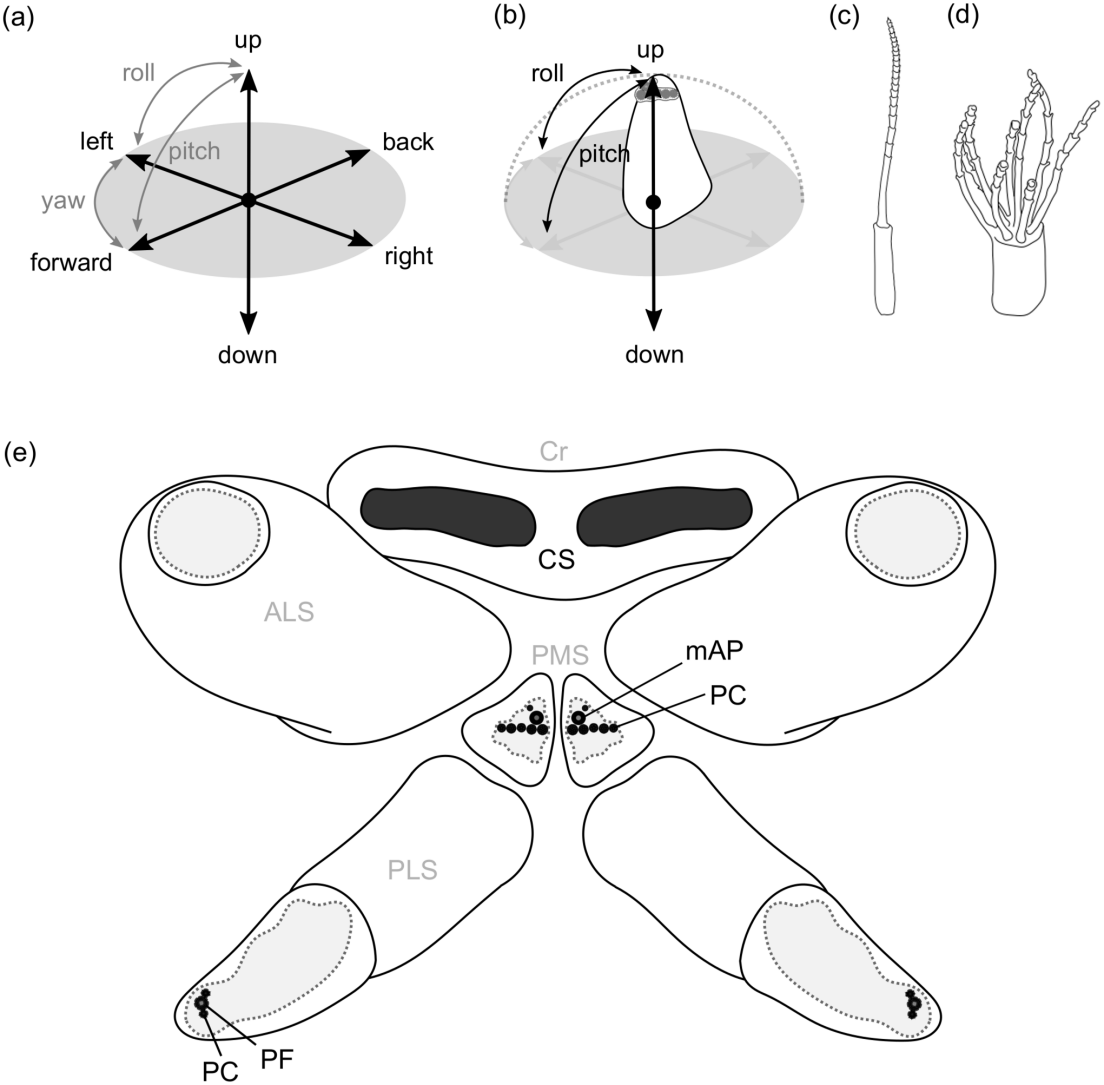
The degrees of freedom of the spinning apparatus of *Badumna longinqua*. **a** In three-dimensional space, a freely movable rigid body possesses six degrees of freedom (DOF) due to translational motion along the axes of an imaginary rectangular cross-axis: up/down, left/right, back/forward and the axis rotations: yawing, rolling and pitching. **b** A posterior median spinneret (PMS) owns one joint with a restricted number of DOF as the spinneret can only move up and down, roll to the left and right and pitch back and forth. Its radius of movement corresponds to a hemisphere. **c** On the PMS two different types of paracribellate spigots (PC) occur: One elongated PC and **d** a row of several PC where multiple shafts originate from one base. **e** The spinning apparatus consists of PMS, anterior lateral (ALS) and posterior lateral (PLS) spinnerets, as well as a pseudo-bipartite cribellum (Cr), containing two separated fields of cribellate spigots (CS) on a common base. Besides several PC, the PMS contain each a minor ampullate spigot (mAP). The PLS contain each a triad of one pseudoflagelliform spigot (PF) flanked by two PC. Spigots involved in the cribellate spinning process are highlighted in black, spigot fields of uninvolved spigots are represented in grey areas. ALS: anterior lateral spinneret, Cr: cribellum, CS: cribellate spigot, DOF: degrees of freedom, mAP: minor amullate spigot, PC: paracribellate spigot, PF: pseudoflagelliform spigot, PLS: posterior lateral spinneret, PMS: posterior median spinneret

Kinematics are also described as the “geometry of motion”. An important aspect of kinematic descriptions includes the degrees of freedom, aside from geometries and velocities to specify all independent possibilities of movement in a physical system. A rigid body in space owns six degrees of freedom along the three axes of an imaginary rectangular cross-axis: Three independent directional movements (translation) and three independent axis rotations (rotation) (Fig. 1a). Any fixation, in a point or plane, reduces the number of degrees of freedom. If several subsystems are connected, e.g., via joints, their individual degrees of freedom are reduced. However, the freedoms of a complete system are the result of an addition of all individual degrees of freedom of each subsystem.

## Results

### Basic kinematics of spinnerets

The simplest way of describing a spinneret kinematically is to consider it a rigid body with only one joint at its base. For this abstraction, we considered a possible elastic deformation of the stiff exoskeleton negligible and the simplification to a rigid body appropriate. Otherwise, the number of degrees of freedom of a flexible body would, from a strictly physical point of view, tend to infinity. With this abstraction of a rigid body respecting only one joint, the radius of movement approximately corresponds to a hemisphere (restricted only by the body of the spider) and depends on the total height of the spinneret (Fig. 1b).

An example of such simple spinnerets are the posterior median spinnerets of the grey house spider *Badumna longinqua*. They are situated between anterior lateral and posterior lateral spinnerets and consisted each of one segment (Fig. 1e). The segments exhibited a pyramidal shape with a flattened apical site bearing different spigots on it (Fig. 1b). A row of paracribellate spigots, involved in the cribellate spinning process, was situated in the middle of a field of other spigot types, not involved in the process (Fig. 1e). The shafts of these paracribellate spigots originated from a shared base, whereas one further but single paracribellate spigots flanked the minor ampullate spigot (Fig. 1c,d). This dyad was situated at the proximal margin of the field of spigots. The mobility of the posterior median spinnerets was restricted in some directions. On an imaginary x- and y-axis, their position was unalterable. However, the z-axis remained for a directional movement so that the spinnerets could move up and down. Translationally, the posterior median spinnerets possessed therefore one degree of freedom. Additionally, only two rotations were possible: “rolling” to the left and right and “pitching “ to back and forth (from posterior to anterior) (Online Resource 1). A “yawing” (a rotation around its own axis), did not occur. As a result, two rotational degrees of freedom, in addition to the translational degrees of freedom, resulted in three degrees of freedom for the posterior median spinnerets in total (Fig. 1b).

**Fig. 2:**
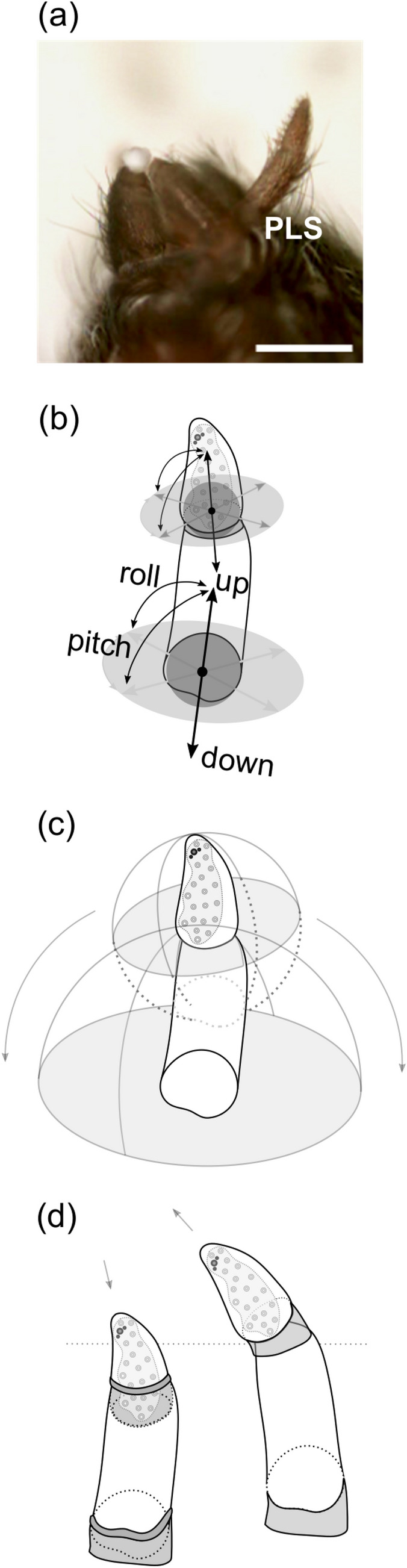
Movement radii of a posterior lateral spinneret. **a** In the anaesthetised spider, the posterior lateral spinneret (PLS) was able to incline almost rectangular to the body; Scale bar 500 µm. **b** The PLS consisted of two joints, which created two axes of inclination. **c** Considering a segmented spinneret with two fixed hinge points the movement range of the basal segment would decrease to a hemisphere according to the length of the basal segment while the range of the terminal segment could, in theory, proximally increase represented by an almost spherical range of movement (dotted line). In reality, the range of movement only corresponded to a further hemisphere. **d** An elastic membrane between the segments provided for a mobile positioning of the hinge points. The range of movement could therefore either be further increased or decreased

In addition to the simple movement of a single joint spinneret, as described for the posterior median spinnerets, the posterior lateral spinnerets were both composed of two segments (Fig. 2). The basal segments were long with a constant diameter and articulated to the body via one joint, respectively. The terminal segments were tapered bearing spigots on their inwards directed sides and articulated to the basal segment by a second joint surrounded by an elastic membrane (Fig. 2b). A pseudoflagelliform spigot flanked by two paracribellate spigots was situated apically on each posterior spinneret (Fig. 1e). The posterior lateral spinnerets could either be adducted by an antero-medial alignment or abducted in postero-lateral direction. During complete abduction, the posterior lateral spinnerets were able to spread from the body to an almost right-angled inclination (Fig. 2a). As could be seen in the video recordings, the two segments could either operate synchronously or independently from each other (Online Resource 2). The posterior lateral spinnerets could, therefore, be, e.g., partially adducted or even further abducted. A second joint between the two segments, thus, created an additional axis of inclination. For the radius of movement, this meant that the hemispherical radius of the basal joint was shortened to the length of the basal segment (Fig. 2 c). The terminal segment could theoretically exhibit an extended range of motion: In contrast to the basal segment, which was proximally limited by a flat body (i.e. the spinneret’s body), the terminal segment mounted the basal segment thus occupying an elevated position on a narrow body. In this case, the range of motion would be proximally limited only by the diameter of the basal segment and would exhibit an approximately spherical shape. This range would not only incorporate the spinneret’s perpendicular configuration but also many further positioning. In reality, the terminal segment never exceeded a perpendicular configuration to the basal segment. The range of movement of the terminal segment corresponded approximately to a hemisphere as well (Fig. 2c). The movement along the z-axis was additionally characterised by the fact that the segments could not only be flexibly extended by stretching the conjunctiva but could additionally be inverted into the apical part of the respective body, thus enabling further positioning by reducing the total length of the spinneret (Fig. 2d). The degrees of freedom of both joints combined, the posterior lateral spinnerets possessed six degrees of freedom (Fig. 2b).

**Fig. 3:**
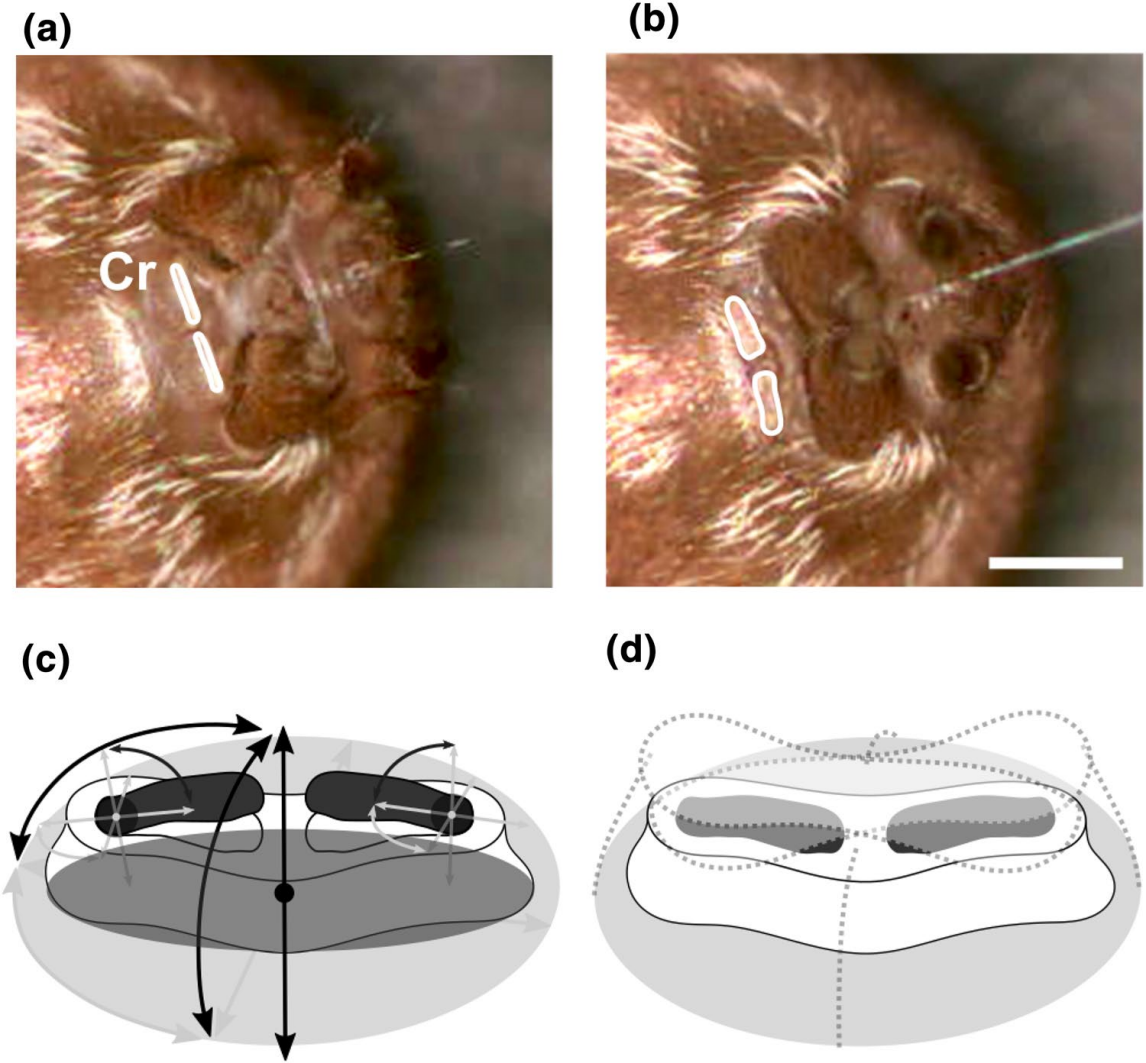
Movements of the cribellum. **a** In the anaesthetised spider, the cribellum (Cr) inclined to posterior or **b** anterior, as well as to lateral directions; Scale bar 500 µm. **c** Due to a fused base, the Cr moved according to one joint with two further angles of inclination enabled by a possible pull out of the right and left spigot fields. **d** The movement range of the Cr corresponded to a flattened hemisphere

The cribellum, anterior of the other spinnerets, consisted of a flat and oval-shaped base on which two separated and elongated fields of uniformly distributed cribellate spigots were placed (Fig. 1e). The shared base of this pseudo-bipartite cribellum could incline in a posterior or anterior direction and lateral direction to the left or right (Fig. 3a, b). Additionally, the cribellum could be either turned outwards or slightly retracted into the body (Online Resource 1). As described for the other spinnerets, this flexibility was achieved by one joint, enabling one-directional movement and two rotations, thus, creating three degrees of freedom. The radius of movement could be represented by a flattened hemisphere (Fig. 3d). Besides, the two fields of spigots exhibited one further rotational movement independent from their base. During the cribellate spinning process, each field was able to bulge out with their medial sides. We illustrated this further movement by two additional hinge points on the lateral margin of the two fields of spigots, which could incline by a few degrees (Fig. 3c). Therefore, a further degree of freedom is assigned to each half of the cribellum.

### Interplay and limits of motion during the cribellate spinning process

**Fig. 4:**
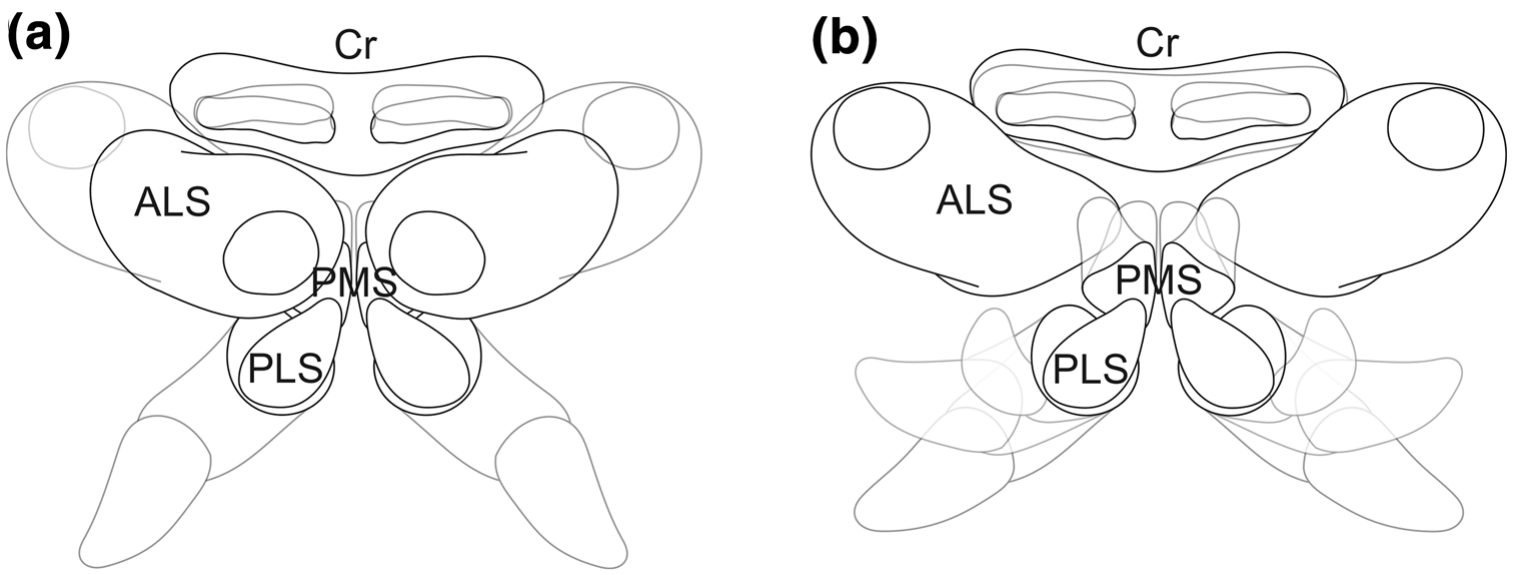
Two-dimensional illustration of the spinning apparatus (ventral view). **a** Most adducted and abducted configurations of posterior lateral (PLS), posterior median (PMS), anterior lateral (ALS) spinnerets, and cribellum (Cr) represented by overlapping contours of the respective spinnerets. **b** During the cribellate spinning process, the ALS rested in an abducted position while PLS, PMS, and Cr moved and occupied different positions shown by transparent contours. ALS: anterior lateral spinneret, Cr: cribellum, PLS: posterior lateral spinneret, PMS: posterior median spinneret

**Fig. 5:**
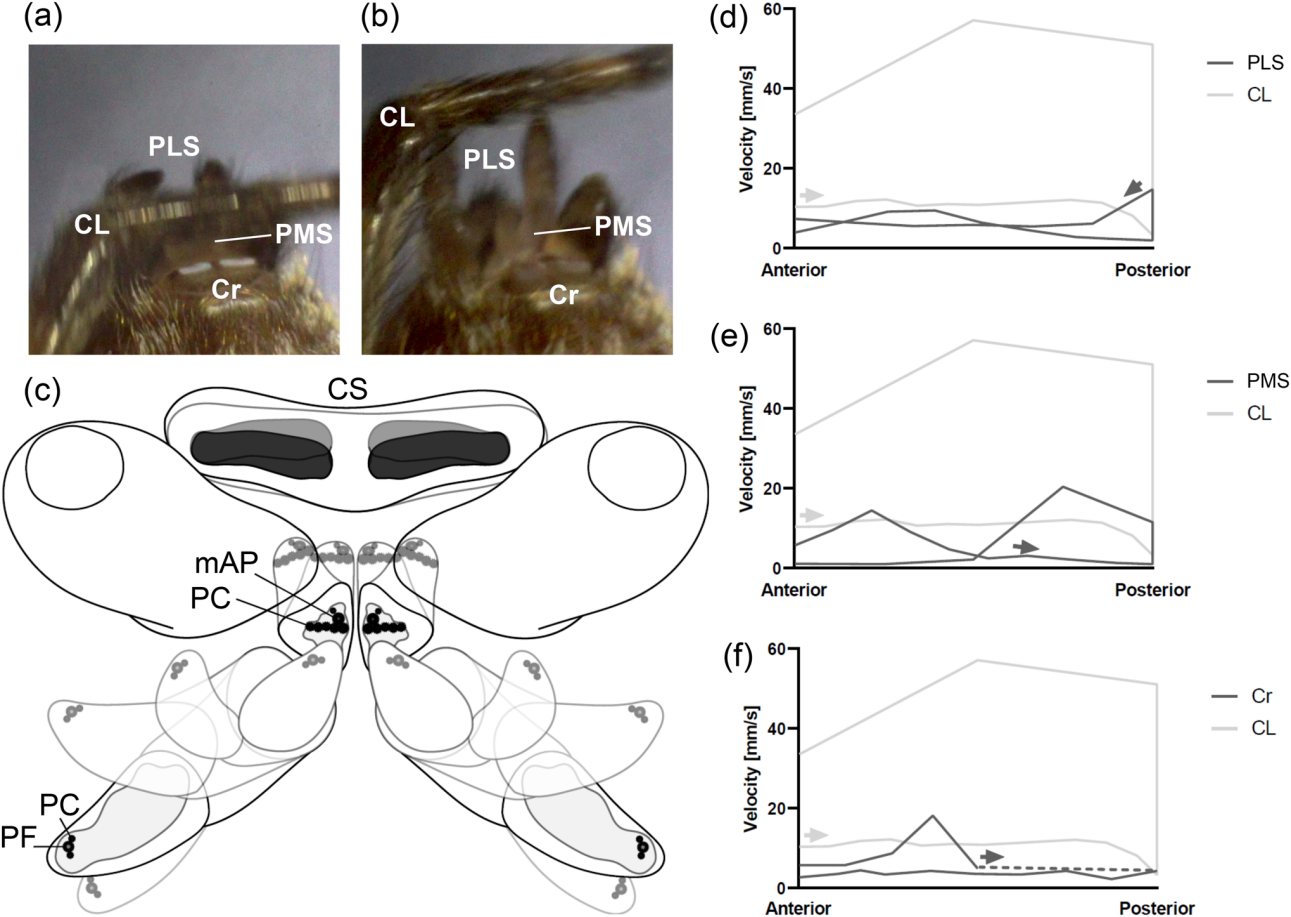
Movements of the cribellate spinning process. **a** During posterior movement of the combing leg (CL), the posterior lateral spinnerets (PLS) moved towards the CL with their terminal segments pointing to anterior; the posterior median spinnerets (PMS) were straightened and followed the movements of the CL; the cribellum (Cr) moved towards posterior. **b** Just before a change of the CL in anterior direction, the PLS were almost straightened and in an abducted posterior position; the PMS moved towards anterior; the Cr inclined to anterior, as well. **c** Model of spinneret movements and spigots involved (spigots involved represented by black dots; spigots not involved represented by the whole spinning field in grey): on the PLS a pseudoflagelliform spigot (PF) was flanked by two paracribellate spigots (PC), during abduction the spigots were far away from the other spigots and spinnerets, during adduction the spigots came into close contact with the spigots on the PMS; on the PMS a row of PC and a single PC flanking the minor ampullate spigot (mAP) were situated; on the Cr two separated fields of cribellate spigots (CS) occurred. **d** Movement velocities of the CL and the PLS during cribellate spinning process: a combing cycle started with the CL at its most anterior position in posterior direction (grey arrow), at the same time the PLS were at a posterior position and moved towards anterior (dark grey arrow). The CL changed direction at its most posterior position returning with a faster movement to its initial position. The PLS returned with a faster movement from its most anterior position in posterior direction, but slowed down and again shortly raised its velocity before the start of a new combing cycle. **e** Movement velocities of the CL and the PMS: at the start of a combing cycle, the PMS were in a medial position and moved towards posterior. At its most posterior position it changed direction with a fast movement and slowed down until it reached its most anterior direction where it returned with a fast movement in posterior direction and slowed down by reaching its initial position. **f** Movement velocities of the CL and the Cr: at the start of a combing cycle the Cr was covered by the CL so that a velocity could not be determined (dotted line). From a posterior position, it inclined to anterior with a uniform movement and returned to posterior again by a sudden movement before reaching its initial position, sample size: **d–f**: (n = 1), (N = 3). CL: combing leg, Cr: cribellum, CS: cribellate spigot, mAP: minor amullate spigot, PC: paracribellate spigot, PF: pseudoflagelliform spigot, PLS: posterior lateral spinneret, PMS: posterior median spinneret

The cribellate spinning process is a highly conserved sequence of movements that could be subdivided into individual repeating cycles (Fig. 5). Each cycle began with a specific spinneret constellation and proceeded in a highly coordinated choreography with each interplay at a specific point in time (Fig. 5c; Online Resource 3). We defined a cycle by the start of the combing leg at its most anterior position, because the spider always started the cribellate thread production at this position (Fig. 5a, d-f). From this position, the combing leg moved in a posterior direction combing over the spinnerets, changed direction back to anterior, and returned to its initial position (Fig. 5d–f). The return movement was not performed if the spider finished the thread production or was interrupted. As already described above for the basic considerations of the individual structures, all spinnerets, and the cribellum could either be completely adducted or abducted independently or occupy any intermediate position (Fig. 4a, b). In contrast to the observation of an individual spinneret, the radii of movement in an interdepended system, consisting of six spinnerets and a cribellum, are mutually limited or do even overlap. During cribellate thread production, the anterior lateral spinnerets remained in an abducted position, thus clearing the way for the movements of the other spinnerets (posterior lateral and posterior median spinnerets), the cribellum (Fig. 4 b) as well as the combing leg bearing the calamistrum.

The posterior median spinnerets started their spinning cycle adducted and in a medial position perpendicular to the body (Fig. 5e). At a velocity of 2.44 mm/s, they first moved in posterior direction by a pitching rotation. This movement occurred at the same time as the posterior movement of the posterior lateral spinnerets so that the posterior median spinnerets were spatially not restricted. Nevertheless, the posterior movement ended after only a slight bending backward to posterior, not using the full possible range of motion. From this position on, the direction of the posterior median spinnerets changed to anterior at a velocity of 11.45 mm/s. Simultaneously, the posterior median spinnerets were abducted and thus spread laterally. Due to the abduction of the anterior lateral spinnerets and their greatest possible clearance, the posterior median spinnerets were able to tilt far forward beyond their initial position and aligned themselves further anteriorly. With a velocity of 1.06 mm/s, the posterior median spinnerets finally returned posteriorly to their initial adducted position again via a change of direction.

The spinning cycle of the posterior lateral spinnerets began in a posterior position with the basal segments being abducted (Fig. 5d). The posterior lateral spinnerets were only slightly tilted backward so that their widest possible radius perpendicular to the body was not exploited. Even though the terminal segments of the posterior lateral spinnerets could theoretically invade the basal parts, e.g., this option was not used during the cribellate spinning process. By tilting inwards, the terminal segments folded slightly towards the basal segment and pointed more and more anteriorly. From this position, the posterior lateral spinnerets moved in almost uniform motion with 6.1 mm/s in antero-medial direction. The anterior movement ended as soon as the basal segment reached a position approximately perpendicular to the body. At that time, the posterior lateral spinnerets approached the posterior median spinnerets closely so that their movements were restricted by the posterior median spinnerets anteriorly. The length of the posterior lateral spinnerets, in addition to their independent segment movements, nevertheless allowed the terminal segments of the posterior lateral spinnerets to protrude above the posterior median spinnerets with their spigots pointing to the spigots of the posterior median spinnerets (Fig. 5c). From this position on, the posterior lateral spinnerets changed their direction and returned to posterior at a velocity of 3.92 mm/s. The terminal segments straightened up during this movement. The posterior lateral spinnerets then remained in a posterior position for a brief moment and folded their terminal segments in a short movement and with a velocity of 14.74 mm/s further back again before finally returning to their initial position.

The cribellum started from an inclined posterior direction and made a smooth anterior movement with a velocity of 4.86 mm/s (Fig. 5f). Approximately from a perpendicular position to the body, it turned its base upwards and directed base and spigot fields frontally to anterior. The medial sides of the spigot fields simultaneously folded upwards so that the cribellate spigots pointed antero-laterally. This faster movement was performed at a velocity of 18.13 mm/s. Immediately after reaching its complete anterior position, the cribellum slowly returned to its initial posterior position.

A detailed observation of the movements revealed that neither the posterior median spinnerets nor the posterior lateral spinnerets or the cribellum used their complete possible range of motion during the cribellate spinning process. In each cycle, the different spinnerets started at specific positions and moved alternately closer together or further apart. Due to a distinct positioning of the different types of spigots involved, the choreography of the spinnerets, nevertheless, accomplished specific contacts at specific points in time and space. The controlled movement enabled close contact between the pseudoflagelliform spigots on the posterior lateral spinnerets and the minor ampullate spigots on the posterior median spinnerets as well as the paracribellate spigots on both spinnerets.

### Capture thread of B. longinqua

**Fig. 6:**
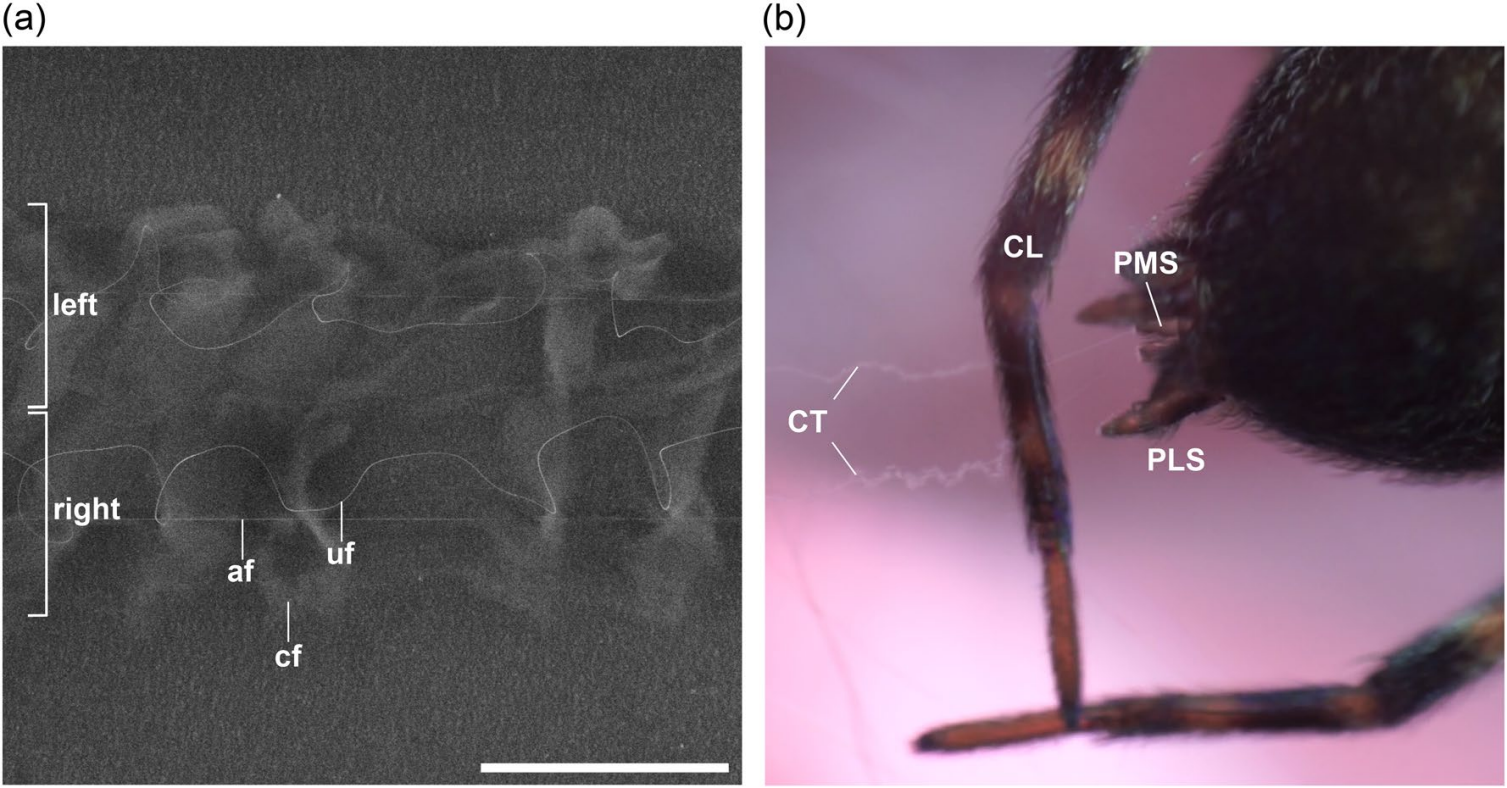
Capture thread of *B.* *longinqua* and its formation. The thread consisted of two strands, which could be either **a** closely parallel or **b** separate. **a** Each strand (left and right half) contained one axial fibre (af), one undulating fibre (uf) and many cribellate fibres (cf) formed into a mat; Scale bar 200 µm. **b** The halves of the cribellate thread (CT) were produced separately right from the beginning. The emergence of individual fibres could be seen at the posterior lateral (PLS) and posterior median (PMS) spinnerets. The final thread structure became visible after processing by the combing leg (CL). af: axial fibre, cf: cribellate fibres, CL: combing leg, CT: cribellate thread, PLS: posterior lateral spinneret, PMS: posterior median spinneret, uf: undulating fibre

The capture thread of *B.* *longinqua*, created during the spinning process, consisted of two separate strands, which were placed either parallel or detached from each other in the web (Fig. 6). The separation resulted from the bilateral arrangement of the spinning apparatus together with the bipartite cribellum, which was particularly evident in the recordings of the spinning process (Fig. 6b). Each of the strands incorporated a single axial fibre (af), an undulating fibre (uf) and a mat of cribellate fibres (cf), which was alternately denser and more loosely packed (Fig. 6a).

## Discussion

Spinnerets move with astonishing manoeuvrability. We studied the kinematics of spinnerets and revealed the spatio-temporal movement possibilities during the cribellate spinning process. The general kinematics of spinnerets, besides, indicate their close relationship to the locomotor system and provide information for other silk-spinning processes. In addition to a transfer to other spinning processes in spiders, this information could also serve as biomimetic inspiration for small-scale fibre assembly.

### Transfer of our model to other spinning systems

The movements observed in the spinnerets of *Badumna longinqua* confirmed a theoretical model of spinneret kinematics very nicely: the range of movement of a one-joint spinneret, such as the posterior median spinnerets, is restricted to a space determined by the length of the spinneret and limited by the body on which the spinneret rests. The movements of the cribellum strongly resemble the flexible movements of a spinneret. A possible evolution from an ancestrally fourth pair of spinnerets has already been suggested due to the different variants of a cribellum (Pechmann et al. 2010). In some species, it has been described that the cribellum can be either undivided with a continuous field of spigots (e.g. in *Uloborus plumipes*) or split into two (pseudo-bipartite; e.g. in *Badumna longinqua*) to several fields of spigots (e.g. in *Dresserus*) (Griswold et al. 2005; Alfaro et al. 2018). We observed that a cribellum possesses almost the same number of translational and rotational movements as a single posterior median spinneret. By an additional bulging of the spigot fields, the cribellum of *B. longinqua* even exhibited a further movement axis on each half of the cribellum. Restrictions due to the merged base of the cribellum are at least partially reduced by this additional mobility. Such a movement has not been described before and could be achieved by either internal musculature or an increase of haemolymph pressure. A multi-joint spinneret, such as the 2-joint posterior lateral spinneret, effects further positioning as several inclinations achieve more combinations of movements. It can be thus concluded that the type and number of joints directly affect the flexibility of a spinneret. However, 2-joint spinnerets are not the most flexible spinnerets that exist: depending on the species, a posterior lateral spinneret possesses up to 4 (e.g. in *Nemesia*), in Mesothelae (e.g. in *Liphistius*), even up to 12 segments with the corresponding number of joints (Marples 1967; Glatz 1973). Besides, most of the anterior lateral spinnerets possess 2 to 3 segments (in *Liphistius* again many more) (Marples 1967; Glatz 1972, 1973). The agility of the spinnerets can, therefore, be refined.

Nevertheless, a limiting factor of spinneret movements is a mutual interference in the overall system (i.e. the spinning apparatus). The spinnerets are grouped very close to each other. If certain action ranges of different spinnerets overlap, space can only be occupied if it is released by another spinneret. The extent to which a restriction is expressed depends on the movement of the adjacent spinnerets at the same time: the further, e.g. the posterior median spinnerets tilt anteriorly, the higher the manoeuvrability of the posterior lateral spinnerets. By protrusion, as possible for the posterior lateral spinnerets to protrude above the posterior median spinnerets, ranges of motion may even overlap without competing. The length of the segments is, therefore, another crucial factor for spinneret movements. During the cribellate spinning process, the abduction of the anterior lateral spinnerets enables free movement of the other spinnerets by clearing possible spatial positions. Due to its flat nature and anterior position in front of the other spinnerets, the cribellum as well is not visibly restricted during the cribellate spinning process. However, during the production of the attachment discs, e.g., the movements of the anterior lateral spinnerets can be restricted anteriorly by the presence of the cribellum (Wolff et al. 2019). As there is hardly any information on spinneret kinematics for other spinning processes, further restrictions caused by spinnerets cannot be excluded. Even a difference in the size proportions of the spinnerets in different species could render certain movements more problematic (Eberhard 2010). However, the general kinematics may already be estimated very well based on the models described here.

### Motoric and sensory control of spinneret kinematics

Besides geometrical descriptions and positions of the spinnerets, the velocity of movements represents an important value to define the kinematics of spinnerets. The velocity of motions during the cribellate spinning process is astonishingly high. During the production of attachment discs in cribellate spiders, the maximum velocity of the anterior lateral spinnerets ranges from 0.6 (e.g. in *Hickmania troglodytes*) to 34 mm/s (e.g. in *Oecobius navus*) (Wolff 2020). Maximum velocities of the spinnerets and the cribellum during cribellate thread production between 11 and 18 mm/s in *B. longinqua* are in the mid-range and seem to be species-specific. We assume the maximum velocity might be restricted to the coordination and manoeuvrability of the spinnerets as well as the combing leg. This raises the question of how muscles move with such a high velocity and how this is controlled (Barth 2020). Usually, muscles of spiders are described to fatigue rapidly after a few seconds of exertion (Bristowe 1932). There are different types of muscle fibres: one that contracts rapidly but fatigues quickly, two which contract slower but for a longer time, and one that remains contracted permanently (Maier et al. 1987; Paul et al. 1991). The long-lasting and fast contraction during the cribellate spinning process could hint to the use of the latter. An increased energetic demand for muscle contraction during spinning could be satisfied by the evolution of respiratory structures as in different lineages within the Araneomorphae the tracheal system shifted towards the spinning apparatus (Ramírez et al. 2020). It has already been described that the spinnerets and their segments can move independently from each other or perform synchronous movements operated by a combination of extrinsic and intrinsic muscles (Whitehead and Rempel 1959; Glatz 1973; Grannemann et al. 2019). Additionally, the cycle always starts at a specific position. This could hint to a constitutional program (e.g. a cascade) of nervous information controlling the movement sequences. It has been described that during dragline production, the spinneret movements and silk release are controlled by several sensilla (mechanoreceptors) which give information about the forces while pulling out the silk and to the orientation of the fibre in space (Gorb and Barth 1996). Similar sensory control of the spinneret movements could be possible by a combination of sensilla on the spinnerets or the calamistrum controlling the cribellate thread production (Foelix and Jung 1978). Furthermore, muscles not only control the movements of a spinneret but define the motion axes of the joints (translation and rotation) according to their length and attachment sites (Glatz 1973; Magalhães et al. 2017). Inside the cribellum, muscles attach anteriorly and posteriorly and thus already indicate possible directions of movement (Glatz 1972). However, a further study would be needed to specify the dynamics of the cribellum as well as the spinnerets.

### The spinneret movements during the cribellate spinning process

The cribellate spinning process is a rhythmical interplay of different spatio-temporal factors leading to a combination of cribellate, axial, and possibly other fibres, such as undulating and supporting fibres (Peters 1984; Joel et al. 2015; Grannemann et al. 2019; Michalik et al. 2019;). This combination of different types of silk affects the mechanical properties of a cribellate capture thread (Michalik et al. 2019; Piorkowski et al. 2020). The spinning sequence is highly conserved, not only in *B.* *longinqua* but also in other cribellate spiders, such as *Uloborus plumipes* or *Kukulcania hibernalis* (Joel et al. 2015, 2016, Grannemann et al. 2019). In *B.* *longinqua*, the movements are all synchronous between the pairs of spinnerets. In other spiders, such as in *K. hibernalis*, the spinnerets can also move asynchronously during cribellate thread production creating another thread geometry (Grannemann et al. 2019). Aside from the movements, the morphology of the spinnerets is crucial for the accessibility of spigots involved (Eberhard 2010; Koebley et al. 2017). The spinnerets can be brought into close contact due to their adapted shape, as also described for *Polenecia producta* (Kovoor and Peters 1988). Besides, the fields of spigots on the terminal segments of the spinnerets are mostly inwards directed, as in the posterior median and posterior lateral spinnerets of *B. longinqua*. During the cribellate spinning process, the apical position of the triad of pseudoflagelliform spigots and two flanking paracribellate spigots on the posterior lateral spinnerets ensures an enlarged radius of movement during an abduction. Thus, the spinnerets function as an extension for the spigot range in this position. During adduction and their most anterior position, however, the spigots of the posterior lateral spinnerets come into close contact with the spigots of the posterior median spinnerets. The posterior median spinnerets on the other hand can approach the cribellum by inclining anteriorly and come into close contact with the cribellum. For the approach of the different structures during the cribellate spinning process, the flexibility of the spinnerets, however, is not fully exploited: only necessary movements are executed and theoretically possible ranges of movements are not completely used. By reducing to essential movements in combination with the required spigot distribution, the spinning apparatus of a cribellate spider can thus achieve different process sequences with one configuration of spinnerets. This not only affects the capture thread production but all the different silk-spinning processes a spider performs.

### Biomimetic inspiration

Studying biological small-scale fibre processing, such as the cribellate thread production, could offer biomimetic inspiration to engineers. Biomimetics, in general, deals with the technical implementation of principles of biological systems. By emphasising necessary conditions which essentially stand for the spinneret movements in the cribellate spinning process, we aimed to find out which conditions can and cannot be neglected for future technical abstractions. Interestingly, it is the spider itself that reduces the scope of movement and processes, as in the cribellate spinning process not all movements of the spinnerets are executed—in comparison to other spinning processes. The course of movement of each spinneret thus takes place along a defined and limited path. This also reduces the possibilities of potential kinematic conditions. For technical implementation, this insight means two things: on one hand, the complexity of the cribellate spinning process takes us to the limits of visualising three-dimensional movements within time. However, this is also a result of the fact that the spinning apparatus compromises due to the compatibility of various process options. On the other hand, the process can be reduced to necessary components of a single function, e.g. the cribellate spinning process. This facilitates biomimetic abstraction by offering scope for optimisation and could inspire the technical fibre spinning of complex systems.

### Conclusion

The movements of the spinning apparatus in the cribellate spider *B.* *longinqua* reveal how specifically the morphological features of a spider’s spinning apparatus can be used to accomplish specific functions. By describing the kinematics of random movements and the regular sequences of movements during cribellate capture thread production, spatial constraints as well as free spaces could be identified. During the cribellate spinning process, multi-joint spinnerets are precisely coordinated for the interweaving of a characteristic multi-fibre system and only appropriate movements are executed in the process. The versatility of spinneret kinematics, combined in a single spinning apparatus, could thus also provide inspiration for the complex spinning of technical fibre systems.

## Supplementary Information

Below is the link to the electronic supplementary material.

Supplementary material 1 (PDF 89 kb)

Supplementary material 2 (AVI 66515 kb)

Supplementary material 3 (AVI 25696 kb)

Supplementary material 4 (MP4 9248 kb)

Supplementary material 5 (EPS 3982 kb)

## Data Availability

Three selected video recordings of the spinneret movements during cribellate thread production and random movements of an anaesthetised *Badumna longinqua* as well as two SEM images of the spinning apparatus are provided as supplemental material accessible in the online version of this publication.
