# Supplementary material for: Cribellate thread production as model for spider’s spinneret kinematics
Source: J Comp Physiol A Neuroethol Sens Neural Behav Physiol. 2021 Jan 23;207(2):127–39. doi: 10.1007/s00359-020-01460-4 (PMC8046689; doi:10.1007/s00359-020-01460-4)
Supplement: Supplementary file 1 — Supplementary material 1 (PDF 89 kb) [file 359_2020_1460_MOESM1_ESM.pdf]

Electronic Supplementary Material:

Cribellate thread production as model for spider's spinneret kinematics

Journal of Comparative Physiology A

Margret Weissbach[1*], Marius Neugebauer[1] and Anna-Christin Joel[1,2*]

1: RWTH Aachen University, Institute for Biology II, Aachen, Germany

2: Macquarie University, Department of Biological Sciences, Sydney, Australia

Corresponding authors (*):

Margret Weissbach, Anna-Christin Joel

RWTH Aachen University, Institute for Biology II

Worringerweg 3, 52074 Aachen, Germany

m.weissbach@bio2.rwth-aachen.de, joel@bio2.rwth-aachen.de

ORCID M.W.: 0000-0003-1879-3048, M.N.: 0000-0003-4962-1660, A.-C.J.: 0000-0002-7122-3047

ESM_1 Ventral view on the spinneret movements of an anaesthetised *Badumna longinqua*, 4x slowed down

ESM_2 Lateral view on the spinneret movements of an anaesthetised *Badumna longinqua*

ESM_3 Ventro-lateral view on the spinning process of *Badumna longinqua* during cribellate thread production, 4x slowed down

ESM_4 Spinning apparatus of *Badumna longinqua* (a) A pair of anterior lateral (ALS) and posterior lateral (PLS) and a pseudo-divided cribellum (Cr) are clearly visible (b) Posterior median spinnerets (PMS) were visible after removal of the ALS; SEM images; Scale bars (a), (b) 300 µm
